# Efficient Feature-Selection-Based Stacking Model for Stress Detection Based on Chest Electrodermal Activity

**DOI:** 10.3390/s23156664

**Published:** 2023-07-25

**Authors:** Ahmad Almadhor, Gabriel Avelino Sampedro, Mideth Abisado, Sidra Abbas

**Affiliations:** 1 Department of Computer Engineering and Networks, College of Computer and Information Sciences, Jouf University, Sakaka 72388, Saudi Arabia; 2 Faculty of Information and Communication Studies, University of the Philippines Open University, Los Baños 4031, Philippines; garsampedro@ieee.org; 3 Center for Computational Imaging and Visual Innovations, De La Salle University, Manila 1004, Philippines; 4 College of Computing and Information Technologies, National University, Manila 1008, Philippines; mbabisado@national-u.edu.ph; 5 Department of Computer Science, COMSATS University, Islamabad 22060, Pakistan; sidraabbas@ieee.org

**Keywords:** wearable sensor, machine learning, stress detection, chest feature, feature extraction, feature selection

## Abstract

Contemporary advancements in wearable equipment have generated interest in continuously observing stress utilizing various physiological indicators. Early stress detection can improve healthcare by lessening the negative effects of chronic stress. Machine learning (ML) methodologies have been modified for healthcare equipment to monitor user health situations utilizing sufficient user information. Nevertheless, more data are needed to make applying Artificial Intelligence (AI) methodologies in the medical field easier. This research aimed to detect stress using a stacking model based on machine learning algorithms using chest-based features from the Wearable Stress and Affect Detection (WESAD) dataset. We converted this natural dataset into a convenient format for the suggested model by performing data visualization and preprocessing using the RESP feature and feature analysis using the Z-score, SelectKBest feature, the Synthetic Minority Over-Sampling Technique (SMOTE), and normalization. The efficiency of the proposed model was estimated regarding accuracy, precision, recall, and F1-score. The experimental outcome illustrated the efficacy of the proposed stacking technique, achieving 0.99% accuracy. The results revealed that the proposed stacking methodology performed better than traditional methodologies and previous studies.

## 1. Introduction

In the current century, wearable equipment has gained importance. Wearable gadgets such as smartwatches, eyeglasses, chest bands, prosthetics, and implants are placed on the human body [1,2,3,4]. Wearable sensors have been used for many healthcare applications such as human activity recognition, stress detection, cognitive health assessment, COVID-19 detection, cardiovascular diseasedetection, human fall detection, Parkinson’s disease detection, etc. [5,6,7,8,9,10,11,12]. Wearable technology is just one aspect of the larger Internet of Medical Things (IoMT) ecosystem [13,14,15,16,17,18]. The IoMT incorporates different medical devices and technologies that can connect online and gather and distribute data for medical purposes. The IoMT includes stationary devices such as hospital screens and imaging equipment, implantable cardiac and insulin pumps, and ambient devices such as smart beds and detectors. Together, these devices collect and transmit data, which can be utilized to monitor patients’ health, identify ailments, and design personalized treatment plans for them [19,20,21]. With wearable technology, precise and durable data estimation is feasible, and this information can be utilized to estimate different factors of human fitness, such as stress status. By collecting information on irregular heartbeats, bedtime habits, and bodily movements, wearable equipment can help individuals manage stress effectively [22,23,24,25,26,27].

Stress-related health issues are becoming more prevalent worldwide and seriously impact people’s mental health and quality of life [28]. Stress is a deadly disease that worsens several dangerous conditions, such as diabetes, cardiovascular disease, and hypertension. The British Health and Safety Executive reported that, in 2021–2022, stress was the main reason for 50% of all occupational diseases [29]. Tension or a powerful sense of anxiety is a sign of distress, a detrimental form of stress. Reduced performance and mental fogginess are side effects of stress. Chronic or severe illnesses can also cause pain that is very difficult for the body and brain to handle, leading to depression and other physical and mental health problems [22]. Short-term stress may not necessarily harm young and healthy people with an adequate protection mechanism in place. Nevertheless, if the disturbing scenario is too often or intense, this might increase the risk of producing pathological diseases associated with stress and depression [30]. A stroke or cardiac arrest can be brought about by short-term stress. On the other hand, long-term stress is known to increase the risk of serious conditions such as coronary artery disease, high cholesterol, diabetes, and obesity [22]. Stress can have a major harmful effect on physical and mental health if it persists. In the biomedical field, self-reported surveys such as the Perceived Stress Scale (PSS) [31] and State-Trait Anxiety Inventory (STAI) are used to measure the psychological perception of stress [32]. Observing physiological responses to stress with detectors is another procedure to quantify the stress status. Pursuing this technique daily for frequent assessment is impossible because it demands time.

Currently, a large number of people are experiencing stress for various reasons. Problems in one’s personal or professional life, an overwhelming workload at work or school, and several other sources of worry are examples of these reasons. Long-term stress exposure can cause severe mental diseases, persistent fatigue, decreased activity, a compromised immune system, and chronic exhaustion. Such disorders can cause sufferers to lose their effectiveness and become ill and even represent a moral and physical threat to others. Employers, educators, coworkers, and friends must be aware of this problem because it could lower productivity among employees and students and lead to a harmful predisposition to other diseases Furthermore, treating such diseases later is more expensive than diagnosing and treating them early. There still needs to be accurate and effective methods for identifying stress. To address this issue, modern technologies such as sensors and machine learning algorithms can save time, money, and human resources. For example, an employer concerned about their employees’ performance should monitor their stress levels, which can lead to reduced productivity and an increased probability of making errors. Implementing machine learning can enhance the quality of diagnostic sensors while reducing the costs of analyses since, with the help of ML, inexpensive and straightforward sensors can outperform expensive ones. Furthermore, detecting overstressed individuals has become highly relevant in light of recent tragic events around the globe.

### 1.1. Motivation

Healthcare is increasingly adopting AI techniques to improve diagnostics, monitoring, and overall patient care. However, the success of AI algorithms heavily relies on the availability of high-quality and diverse datasets [20,33,34,35]. In the case of stress detection, access to large-scale and labeled datasets is limited, which hinders the development and evaluation of accurate AI models. Our study sought to contribute to the availability of such datasets by utilizing the Wearable Stress and Affect Detection (WESAD) dataset. By conducting experiments and analyzing this dataset, we aimed to provide valuable insights into stress detection using chest-based features. The dataset consists of physiological signals, motion sensors, and self-reported labels obtained from devices worn by participants in various stress-inducing scenarios. By leveraging the WESAD dataset, we can explore the potential of chest-based features in stress detection. These features include the heart rate, respiration, and electrodermal activity, which are relevant indicators of the stress level. Through our analysis, we aimed to uncover patterns, correlations, and discriminative features that can aid in accurately detecting stress using AI methodologies. Different researchers have proposed different techniques to predict stress, such as sensor-based approaches [36] and ML and DL approaches, such as KNNs, RF, Adaboost [37], and CNNs [38], but these studies are limited in their performance. To address all these problems, this research proposes an approach using a machine learning algorithm including logistic regression (LR), linear discriminant analysis (LDA), quadratic discriminant analysis (QDA), and a stacking model by applying chest features from the WESAD dataset.

### 1.2. Contribution

This study makes stress detection using chest-based data more precise and efficient. The paper’s primary contributions and distinguishing characteristics are listed below:

- The research presents a stacking model based on three machine learning algorithms (LR, LDA, and QDA) for predicting stress using data from chest-worn sensors.
- The WESAD dataset, which includes the five states of transitory, baseline, stress, amusement, and meditation, was turned into a suitable format for the proposed framework. Next, we performed feature analysis and selected the optimal features based on statistical measures.
- The observed outcome illustrated the efficacy of the proposed stacking technique, achieving 0.99% accuracy. The results revealed that the proposed stacking methodology performed better than traditional and previous studies.

### 1.3. Organization

Section 2 provides the most recent relevant research on wearable sensor-based methodologies, ML, and approaches in the healthcare industry. The proposed approach is discussed in Section 3, which also addresses dataset visualization, data preprocessing, RESP features, data collection, feature analysis (Z-score, feature selection, SMOTE, and normalization), and machine learning algorithms. Section 4 describes the proposed approaches’ evaluation measurements, results, and findings. Finally, Section 5 concludes the research work and offers suggestions for future investigations.

## 2. Related Work

This section presents the background of previous state-of-the-art (SOTA) techniques used to predict stress, such as wearable-sensor-based, machine learning, and deep learning approaches.

### 2.1. Wearable-Sensor-Based Methodologies

A major issue with ambient assisted living technologies is automated stress detection. The authors of [36] discuss the findings of two studies that used a chest belt-mounted pacemaker to identify stress. The device verification trial determined the sensor’s dependability by comparing parameters recorded by the belt and heart rate data to data obtained by the gold standard apparatus. They chose highly correlated, low average error data segments of significant measurements of chest data for additional processing utilizing an explicit synchronization and data cleaning technique. The clinical study’s strategy contained two steps that lasted for 10 min: a palliative step and a mentally stressful stage. They created a straightforward technique for identifying stress by operating three-time domain parts of the heart rate motion. According to the results of two state-of-the-art methods used to analyze the exact data, the strategy produced results with an accuracy, sensitivity, and specificity of 74.6, 75.0, and 74.2 percent, respectively. In article [39], the relationship between pain and stress is discussed, as well as methods for measuring and identifying them with the aid of diagnostic implants and worn sensors. Wearable sensors monitor physiological indications, including pulse rates, neural actions, muscle movements, electrodermal activity, breathing speeds, blood volume pulsation, and skin conductance. The authors aimed to develop a wearable health service system technique for stress and pain inspection by examining the wearable detectors used in healthcare equipment.

The proposed architecture in [23] was established on attribute extraction from gyroscopic measurements and encourages inexpensive wearable sensors. Heart rate variability (HRV) characteristics and cardiac timing intervals comprise the feature space for assessing a disease’s severity. Modern machine learning (ML) techniques divide severity levels into mild, moderate, and severe categories. With an F1-score of 94.29 percent and an accuracy of 94.44 percent, Light Gradient-Boosted Machine (Light GBM) performed the best. Moreover, evaluations based on game theory were used to study the top attributes and how they generally affect the severity level. The most typical characteristics of AS severity are the isovolumetric contraction time (IVCT) and isovolumetric relaxation time (IVRT).

To further broaden our paper’s scope, we studied relevant works that have explored similar areas in healthcare and AI. The authors of [40] addressed security and authentication challenges in wireless medical sensor networks, which are relevant in ensuring the integrity and confidentiality of data collected from wearable devices. In [41], the authors proposed a fog computing architecture that leverages software-defined networking (SDN) to enable efficient and secure data processing in healthcare applications. This work is relevant as it highlights the importance of infrastructure and networking solutions to handle the increasing volume of data generated by wearable devices and healthcare IoT systems. The authors of [42] presented a knowledge-infused learning framework for cardiovascular event diagnosis. Although the focus of this work is different, it showcases the potential of AI techniques in healthcare and highlights the importance of developing accurate and reliable models for medical diagnostics. Information security has received attention from academic and industrial sectors for data prevention, integrity, and modification. Traditional and mathematical security models address information-related challenges, although they do not guarantee 100% data privacy. Computational intelligence is a powerful technology that draws inspiration from biological evolution. It is an intelligent agent that recognizes patterns in complicated and real-world contexts. Artificial neural networks, fuzzy logic, evaluation computation, and hybrid methods are other subcategories of computational intelligence. Each branch of computational intelligence was examined in [43] from the cybersecurity perspective, along with their benefits and drawbacks.

### 2.2. Machine and Deep Learning Methodologies

Study [37] aimed to identify stress in individuals using machine learning techniques to enhance their quality of life. The WESAD dataset, a publicly accessible multimodal dataset, was utilized to access different ML methodologies for identifying individual stress via ML methods such as k-NN, linear discriminant analysis, random forest, AdaBoost, and Support Vector Machine. The random forest algorithm performed more adequately than other algorithms to classify two and three categories, with values of 83.34 and 65.73 regarding the F1-score. In our comprehensive review, we focused on stress recognition using wearable detectors and appropriate machine learning approaches. This analysis looks at how stress can be detected using wearable detectors, photoplethysmography (PPG), electrocardiograms (ECG), electroencephalograms (EEG), and other sensing devices in a variety of situations, including driving, learning, and working [22].

We proposed an approach based on a convolutional neural network multi-level DNN with hierarchical learning abilities. A hierarchy of networks is trained to use multivariate time-series data from wrist-based and chest-based device bio-signals to create high-level features for each bio-signal feature. The high-level features are incorporated into one coherent presentation using a proposed model-level fusion technique, which divides the stress states into baseline, stress, and amusement categories. The WESAD dataset for cognitive health is employed to assess the methodology, which corresponds well with cutting-edge techniques and has an outstanding interpretation accurateness of 87.7% [38]. The author of this study designed a DNN strategy that comprises a multilayer perceptron (MLP) neural network and a one-dimensional CNN. Deep neural networks can extract features from raw data through the neural network’s layers without the need for manually created features. To complete two tasks, the deep neural networks examined physiological data obtained from wrist and chest sensors. Each neural network was developed to interpret wrist or chest sensor data. The networks’ first objective was to distinguish between stressed and non-stressed states in a binary classification for stress detection. The networks used a three-class classification scheme in the second experiment to distinguish between baseline, stressed, and amused conditions. The networks were prepared and evaluated using data from earlier studies made publicly available.

Regarding the classification accuracy for binary and three-class classification, the deep convolutional neural network achieved 99.80% and 99.55%, respectively. The deep MLP neural network attained 99.65% and 98.38% accuracy rates for binary and three-class classification, correspondingly [44]. The authors of [28] proposed a new wearable gadget that concurrently estimates electroencephalograms (EEG) and electrocardiograms (ECG) using a non-invasive method. This strategy combines an analog front end (AFE) with a digital back end (DBE) processor based on machine learning to predict mental stress utilizing just three electrodes. With the use of readily available commercial components, a PCB prototype was created. The created prototype has a classification accuracy of 92.7%, a reasonable noise performance of 0.1 Vrms, and can forecast mental stress. The suggested method is portable and straightforward to wear (behind the ear). For several stress scenarios, including the Stroop Color and Word Test and the Arithmetic Test, data were collected from 25 subjects. An external neural network (SNN) classifier categorized the stress states using various EEG- and ECG-based feature combinations.

The authors of [21] presented a thorough study on stress detection, beginning with an initial investigation including a population of frail older adults with mild cognitive impairment (MCI) who took part in mental and motor rehabilitation sessions, were fitted with wearable physiological sensors, and were given a smartphone application for physiological tracking. Data were gathered using replies received during therapy sessions to determine how physical activity favors cognitive training. Machine learning classifiers were used for the prediction of stress utilizing real-world data. In [45], the authors used a machine learning algorithm to diagnose depression, anxiety, and stress by gathering data using questionnaires from employed and unemployed people from different countries. Five distinct ML algorithms were used to predict the occurrence of anxiety, sadness, and stress on various severity levels. These algorithms are extremely accurate; thus, they are well suited to forecasting psychological issues. Classes were determined to be imbalanced in the confusion matrix after using various approaches. To help choose the random forest classifier as the highest accuracy model among the five applied algorithms, the F1-score metric was included. The authors of [46] suggested SELF-CARE, a wrist-based stress detection technique that uses context-aware selective sensor fusion and dynamic sensor data-driven adaptation. The proposed approach learns to change the fused sensors in the context of the system using motion, enhancing the performance while preserving energy. In the publicly accessible WESAD dataset, SELF-CARE offers a cutting-edge performance, with accuracy scores for the three-class and two-class classification problems of 86.34% and 94.12%, respectively.

## 3. Proposed Model

**Figure 1:**
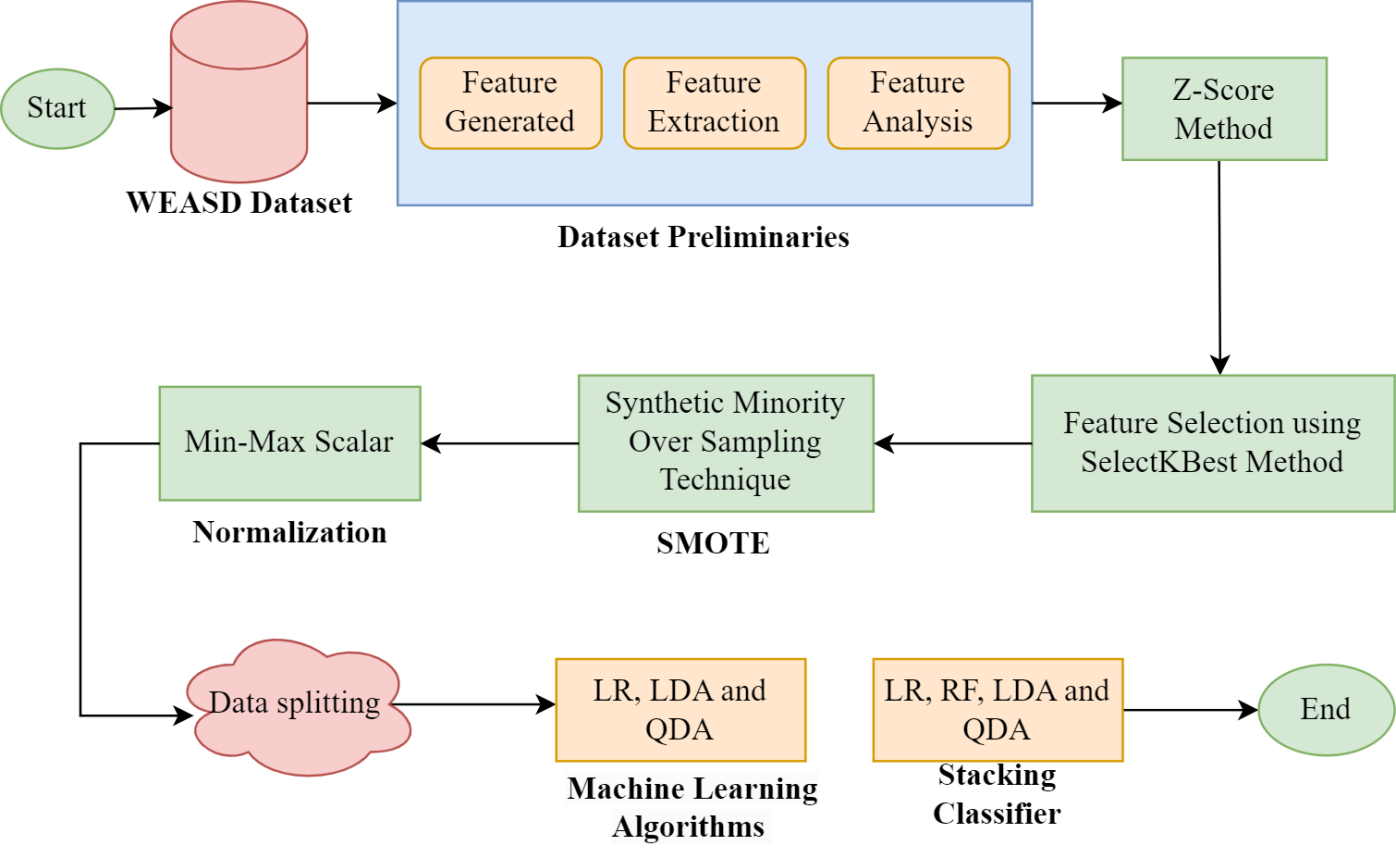
Proposed framework overview for stress detection.

The steps of the proposed approach are described in this section. Machine learning algorithms are utilized for chest-feature-based stress prediction. The proposed methodology is assessed on the following evaluation metrics: precision, accuracy, recall, and F1-score. The research was validated on Anaconda using jupyter notebook and Python language. Figure 1 illustrates the steps of the proposed work individually. Firstly, we used the publicly available WEASD dataset and performed exploratory data analysis steps for data visualization and preprocessing to convert raw data into a helpful format. RESP was utilized to extract useful features, and after extracting them, we generated 28 data frames from 14 subjects. In the data preprocessing steps, first, we applied the Z-score to remove outliers from the dataset. Then, the feature selection step was carried out using the SelectKBest technique for selecting features. At last, SMOTE was applied for imbalanced datasets and normalization was applied to scale the feature values in a specified range. The first ML classifier (LR, LDA, and QDA) was individually applied to the preprocessed dataset. Then, a stacking technique based on RF, LR, LDA, and QDA was applied to improve the performance.

### 3.1. Dataset Preliminaries

This research utilized a dataset available on the public UCI machine learning repository, which was proposed in [47]. The Wearable Stress and Affect Detection (WESAD) dataset is widely used in stress detection. It consists of physiological signals, motion sensors, and self-reported labels collected from wearable devices worn by participants in controlled experiments. Our study specifically focuses on the chest-based features available in the WESAD dataset. These features include heart rate, respiration, and electrodermal activity. These physiological signals have been widely studied in the context of stress detection and have shown promise in accurately capturing stress-related responses in the body. The heart rate provides information about the cardiovascular stress response, while respiration patterns can indicate changes in the autonomic nervous system. The electrodermal activity, measured through skin conductance, reflects the electrical properties of the skin and is known to be sensitive to emotional arousal, including stress.

A RespiBAN professional chest-worn device was used for data collection. The RespiBAN has sensors for measuring ACC and RESP and can serve as an intersection for up to four other modalities. The four analog ports record the ECG, EDA, EMG, and TEMP. At 700 Hz, all signals are captured. The RespiBAN covers the subject’s chest. A respiratory inductive plethysmograph detector is employed to document the RESP. The usual three-point ECG is used to record the ECG data. The EDA signal is captured on the rectus abdominis, and the TEMP sensor is positioned on the sternum to permit the subject to move as much as possible. The upper trapezius muscle’s EMG data are logged on both sides of the spine. The collected data are kept locally and then moved to a computer for additional processing after the experimentation to prevent wireless packet loss. These were the steps performed in [47] to collect data. This research uses this dataset to detect stress using the RespiBAN chest sensor device data.

Since we have the raw data from the chest sensor, we created a feature to obtain valuable data. To this end, we used EDA, EMG, and TEMP columns from the chest sensor’s raw data. To divide the raw signals into one-minute windows for this feature generation procedure, we employed a sliding window with a window shift of 0.25 s (except for EMG data, which was processed with a 5 s window). We started with the TEMP data, representing temperature in degrees Celsius. We produced several fundamental characteristics for this column, such as the mean value, standard deviation, dynamic range, and slope for each window.

**Figure 2:**
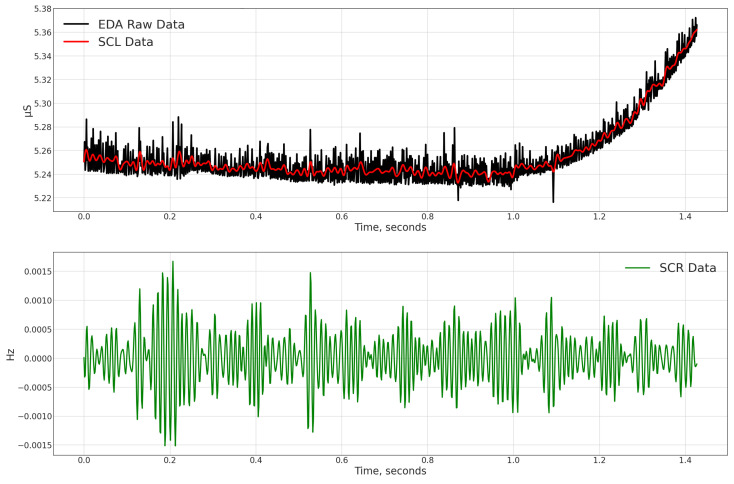
Extracting SCR and SCL from EDA raw data.

**Figure 3:**
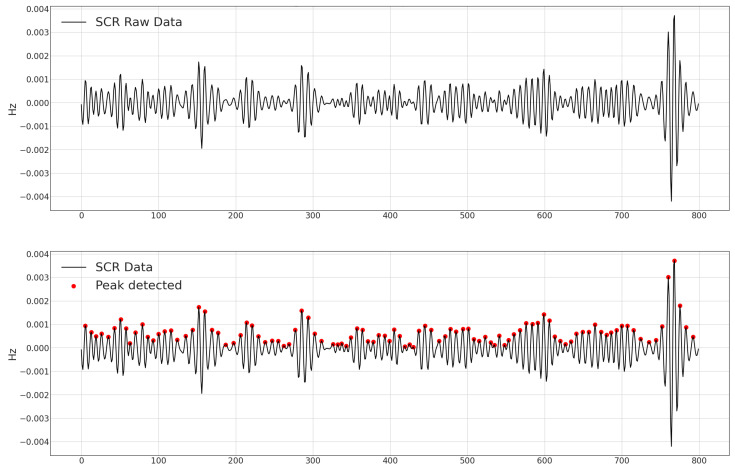
SCR data peak detection.

Next was the EMG data, which contained electromyography readings calculated in mV. As stated, a unique 5 s processing window was used for this feature. The mean value, standard deviation, and dynamic range for each window were generated, along with the same characteristics as the temperature column. The EDA data, or electrodermal activity as measured in S, were processed last. Using the raw data, we calculated each window’s mean value, standard deviation, dynamic range, and slope. We divided EDA into SCL and SCR, which we found after performing some investigations. The Skin Conductance Level (SCL) and Skin Conductance Responses (SCRs), caused by sympathetic neural activity, are vital components of the EDA complex. Hence, we generated mean and standard deviation features for SCL and SCR components. The number of peaks for each window is another intriguing feature we evaluated for the SCR component. Figure 2 illustrates the features extracted from the EDA raw data, and Figure 3 illustrates the SCR peak data detection. The additional column from the raw data is called ACC, which contains the accelerometer data utilized to characterize the movement. We created the following characteristics from this data using ACC data, such as Max|ACC|, 3D means, and 3D standard deviation for all axes. The absolute integral depicts movement on all axes and in three dimensions. This was generated within a window of 5 s. The window shift remained constant at 0.25 s. Electrocardiography data in the dataset are represented in the ECG column, measured in mV. To add behavioral heart features, we generated the following attributes: heart rate mean values, standard deviation, maximum and minimum values for every window, NN50 feature, RMSSD feature, average and standard deviation values of distance among peaks and energy in diverse frequency bands, and rate feature.

From a non-specialist in life sciences point of view, this feature describes different biological heart effects, so we accept this data as they are. Window parameters were standard. Here, to find distances between peaks, we used the find_peaks algorithm to find the peaks and used the FFT transform of diff(diff(peaks_places)) to generate feature energy in the different frequency bands. We smoothed the plot with the savior filter and used a lowpass filter.

### 3.2. RESP Features

Respiration (RESP) features were produced to understand the effect of stress on the breathing process of a patient. The features are mean and std inhalation (I) duration; max amplitude, mean, and std exhalation (E) duration; and max amplitude, E ratio, mean, and standard deviation values of analog of volume and respiration rate. To generate these, we used the standard window parameters described above. To determine the duration of breathing, we used:

- The find_peaks mechanism. It gives very good results, but sometimes there were several non-detected peaks.
- As a solution to this problem, we proposed an algorithm called find_duration, which finds the duration only in places where real respiration is detected without error.
- After this algorithm usage, the amplitude can be easily determined.
- As a volume analog, we used the absolute integral of RESP sensor values.

### 3.3. Data Collection

In this research, we focus on chest sensor data, generally the features extracted from chest sensor data with some adaptations made based on the raw data parameters such as the sampling frequency. After extracting the features, we generated 28 data frames from 14 subjects for the chest-based dataset.

### 3.4. Feature Analysis

Further, we created a feature analysis function that creates binary target values and builds density distribution functions. We used it to analyze features and understand which ones can be dropped before fitting. We analyzed data from one subject and had great results. We could separate stress states and calmness with an accuracy of about 100% for a particular subject. Moreover, after analysis (density plots), the below features cannot help separate these classes. Thus, we decided to drop them. We determined that there are too many data for fitting. Thus, we decided to make a large test dataset to make fitting faster.

#### 3.4.1. Z-Score Method

The Z-score method is a commonly used statistical technique for identifying and removing outliers from a dataset. It is based on standard deviation and measures how far away every data attribute is from the mean regarding the standard deviation [48]. In our scenario, outliers were removed from the dataset if determined to be data entry errors or anomalies, and the outliers were replaced with missing values (NaN) to retain the dataset’s length and structure but exclude extreme values from the analysis. The outliers were replaced with more reasonable values based on domain knowledge or advanced imputation techniques if the outliers represented genuine data points.

#### 3.4.2. Feature Selection Using SelectKBest Method

The feature selection strategy called the SelectKBest approach was used to pick the K-best features from a dataset using statistical criteria. It evaluates the association between every feature and the target variable and assigns a score to every feature. The feature is often considered for the target variable, resulting in a higher score [49]. In our scenario, we assume that DataFrame X contains the features and a numpy array or pandas Series y contains the target variable. The score_func parameter is set to f_classif, which is appropriate for classification tasks. After fitting the selector to the data, the transform method transforms the dataset X to include only the selected features. Then, we accessed the indices of the selected features using get_support(indices=True) and retrieved their names from the original feature set. The number of features chosen, i.e., 10, determines the value of k.

#### 3.4.3. Synthetic Minority Over-Sampling Technique (SMOTE)

SMOTE is a popular technique used in machine learning to address class imbalance problems in classification tasks [50]. It is specifically made to deal with imbalanced datasets where the majority class has a disproportionately small number of instances compared to the minority class [51]. In our scenario, X represents the feature matrix and y represents the target variable. The fit_resample method performs SMOTE oversampling, and it returns the resampled feature matrix X_resampled and the corresponding target variable y_resampled.

#### 3.4.4. Normalization

Normalization, or feature scaling, is a preprocessing approach utilized in ML to adjust different features or variables to a similar scale [52,53,54]. It is performed to ensure that no particular feature dominates the learning algorithm due to its larger magnitude or unit of measurement. Normalization typically involves transforming the values of numerical features to a standard scale, usually ranging between 0 and 1 or −1 and 1. There are several standard methods for normalization; in this research, we utilized Min-Max normalization or rescaling. This method scales the feature values to a specified range, often between 0 and 1. The formula for Min-Max scaling is illustrated in Equation  (1):

(1) $$x_{normalized}=\frac{\left(x-min(x)\right)}{\left(max(y)-min(y)\right)}$$

By employing these techniques, we aim to preprocess the data, extract relevant features, handle class imbalance, and normalize the data for subsequent analysis and modeling. Each technique was chosen based on its suitability for stress detection, the previous literature on stress-related features, and best practices in data preprocessing and machine learning. These methods enhance our stress detection models’ accuracy, interpretability, and robustness. Then, we split the dataset into training validation and testing sets.

### 3.5. Machine Learning Classifiers

Three ML classifiers, such as LR, LDA, QDA, and one ensemble stacking model, are used to predict stress problems using chest-based sensor data.

Logistic Regression: Logistic regression is a statistical analytical method that utilizes prior dataset observations to forecast a binary output, such as yes or no. A logistic regression algorithm constructs predictions regarding a dependent data variable by examining the correlation among one or more independent variables that are already present [55].

Linear and Quadratic Discriminant Analysis: Linear discriminant analysis (LDA) is a technique for reducing dimensionality. It is a pre-processing phase in machine learning and feature classification applications [56]. LDA is employed when a linear border among algorithms is essential, and QDA is utilized to determine a non-linear boundary among algorithms. When the feedback categories are different, and the distribution of X = x for each class is typical, LDA and QDA perform similarly. Stacking: A stacking classifier is used to leverage the strengths of different models and improve the overall prediction performance. It can help confound the restrictions of separate models and deliver more authentic and strong predictions. Stacking is a flexible and powerful technique but it requires careful consideration of a model, feature engineering, and avoiding overfitting. It can be a practical approach for improving the classification performance when used appropriately [57]. In this experiment, we used a stacking classifier by combining the predictions of multiple base classifiers and another classifier, referred to as the final_estimator, to make the final prediction. The following base classifiers and final estimator are used:

- Quadratic Discriminant Analysis (QDA): Quadratic discriminant analysis is a classification algorithm that assumes each class follows a quadratic distribution. It estimates class boundaries based on the quadratic discriminant function.
- Linear Discriminant Analysis (LDA): Linear discriminant analysis is a classification algorithm that assumes each class follows a Gaussian distribution. It calculates the optimal linear discriminant functions to separate the classes.
- Logistic Regression (LR): The classification procedure known as logistic regression uses the logistic function to model the connections among the input variables and their possibility of belonging to a certain class.
- Random Forest: Random forest is an ensemble learning method for making predictions incorporating numerous decision trees. The final forecast is obtained by voting after each tree in the forest has been trained using a random portion of the training data.

The choice of the stacking model in our proposed approach is based on its potential to improve the overall performance and robustness of the stress detection system. The stacking model is an ensemble learning technique that combines multiple base models to make predictions. It aggregates the predictions from different models, effectively leveraging the strengths of each model. By combining the outputs of multiple models, the stacking model aims to capture diverse perspectives and improve the overall predictive power.

Algorithm 1 describes the method of predicting stress using the chest sensor dataset. The input is the dataset and the output is the model performance. The algorithm consists of several steps, such as data visualization ( $D_{v}$ ) to visualize the data to gain insights into their structure and relationships. Data preprocessing ( $D_{p}$ ) is performed to clean and transform the data to make them suitable for modeling. After that, the RESP feature ( $R_{f}$ ) step involves extracting features from the data related to respiratory behavior. Feature analysis ( $F_{A}$ ) is performed, in which the following steps are carried out: Z_Score Method, SelectKBest Method, SMOTE for balancing data, and standard scaler normalization. The dataset is split into training and testing sets. Four classifiers are trained on the training set: LR, LDA, QDA, and the stacking model. The classifiers are evaluated on the testing set using evaluation metrics. The algorithm returns the best results from the evaluation of the classifiers.
**Algorithm 1** Algorithm for Stress Prediction.
1:
Input : Chest Sensor Dataset $C_{d}s$
2:
Output : Model Performance $M_{p}$
3:
$D_{v}\leftarrow$ Data Visualization4:
$D_{p}\leftarrow$ Data Preprocessing5:
$R_{f}\leftarrow$ RESP Features6:
$D_{C}\leftarrow$ Data Collection7:
$F_{A}\leftarrow$ Features Analysis8:   
Z_s← Z_Score Method9:   
F_s← SelectKBest Method10: 
S← SMOTE for balancing data11: ← Min_Max Scalar12:
$x_{train},x_{test},,y_{train},y_{test}$ {Train Test split}13:
$Classifiers_{ML}$
14:LR ←Logistic Regression15:LDA ←Linear Discriminant Analysis16:QDA ←Quadratic Discriminant Analysis17:Stacking ←Stacking Ensemble Model18:
E_m← Accuracy, Precision, Recall, F1_Score {Evaluation metrics}19:Return ← Best Results


## 4. Experimental Results and Discussion

This research validation uses the WESAD dataset accessible at the public UCI machine learning archive. This section explains the evaluation measurements used for the experiment result and model discussion. It also provides feature extraction, feature analysis, and feature selection techniques on the used dataset. It applies the stacking technique by combining three machine learning algorithms to improve the model’s performance.

### 4.1. Evaluation Metrics

The experiment evaluation is examined using accuracy (A), F1-score (F1), recall (R), and precision (P) measurements. These evaluation measurements estimate how sufficiently the proposed approach performs. The percentages of false positives (FP), true positives (TP), and false negatives (FN) are calculated to evaluate the proposed model’s precision. The accuracy estimate is represented in Equation (2). It measures the actual positives as a percentage of all positive data and is sometimes referred to as a value that is greatly anticipated. The precision rate is shown in Equation (3). Sensitivity, the probability of prediction, and the possibility of a true positive represent the ratio of real positives to TP and FN in a dataset. Equation (4) shows the recall rate. The F1-score is calculated as the weighted average of recall and precision. Equation (5) provides the F1-score.

(2) $$Accuracy=\frac{TP+TN}{TP+TN+FP+FN}$$

(3) $$Precision=\frac{TP}{TP+FP}$$

(4) $$Recall=\frac{TP}{TN+FN}$$

(5) $$F1-score=2\times \frac{Precision+Recall}{Precision+Recall}$$

Table 1 illustrates the outcome of a proposed model, including all evaluation metrics for every model. The models evaluated are LR, LDA, QDA, and stacking. LR attained an accuracy of 0.978, a precision of 0.998, a recall of 0.975, and an F1-score of 0.986. LDA attained an accuracy of 0.955, a precision of 0.999, a recall of 0.945, and an F1-score of 0.971. QDA attained an accuracy of 0.978, a precision of 0.998, a recall of 0.975, and an F1-score of 0.986. Stacking attained an accuracy of 0.997, a precision of 0.999, a recall of 0.997, and an F1-score of 0.998. These results suggest that all models perform well, but their performances differ. LR, QDA, and stacking have similar scores, with a high accuracy, precision, recall, and F1-score. LDA has slightly lower scores, indicating a slight deviation from perfect predictions compared to the other models.

**Figure 4:**
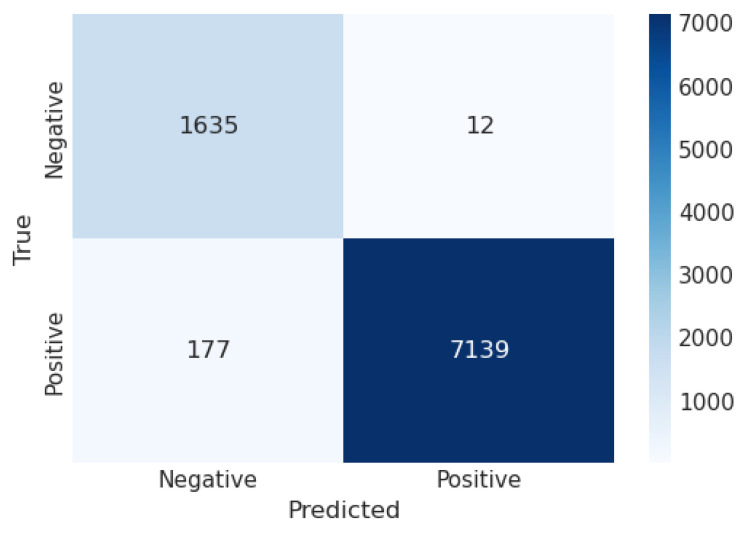
Confusion matrix of logistic regression.

**Figure 5:**
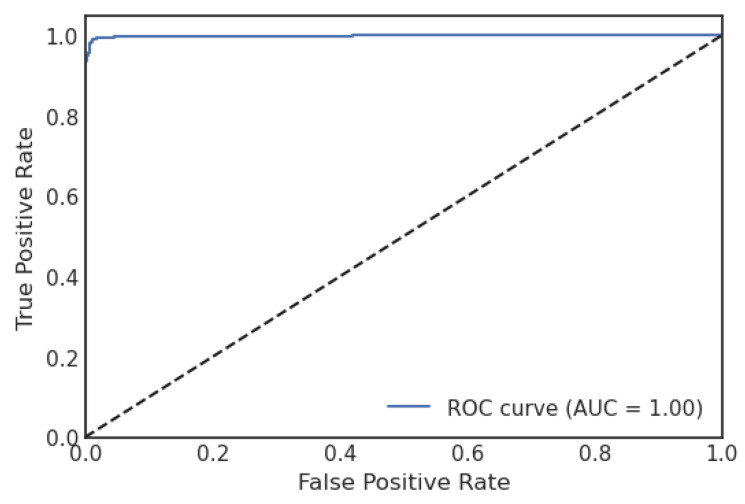
ROC curve of logistic regression.

A confusion matrix (CM) defines how sufficiently a classification algorithm performs. A CM illustrates and aggregates a classification algorithm’s performance. Figure 4 shows the CM of the LR algorithm of the proposed model. It shows that if the TP and TN values are greater than the FP and FN values, the performance of the proposed model improves, and the model performs well on the used dataset. Figure 5 illustrates the proposed model’s recursive operating characteristics (ROC) graph. The model outperforms, with an area of the ROC curve of 0.997%.

**Figure 6:**
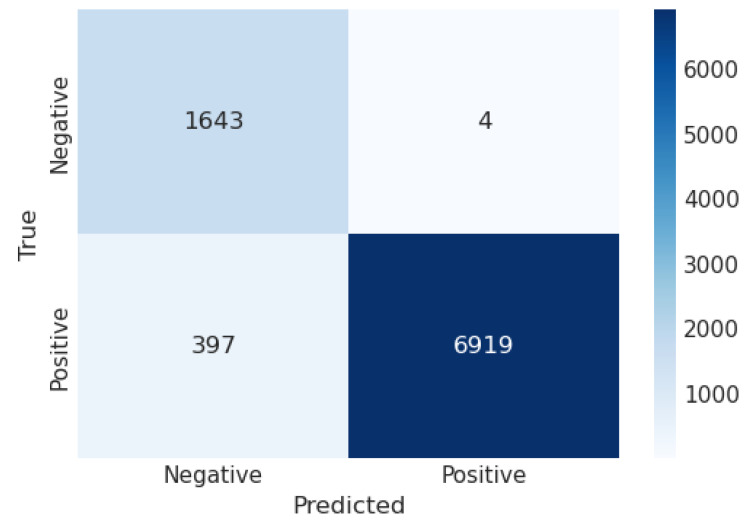
Confusion matrix of LDA.

**Figure 7:**
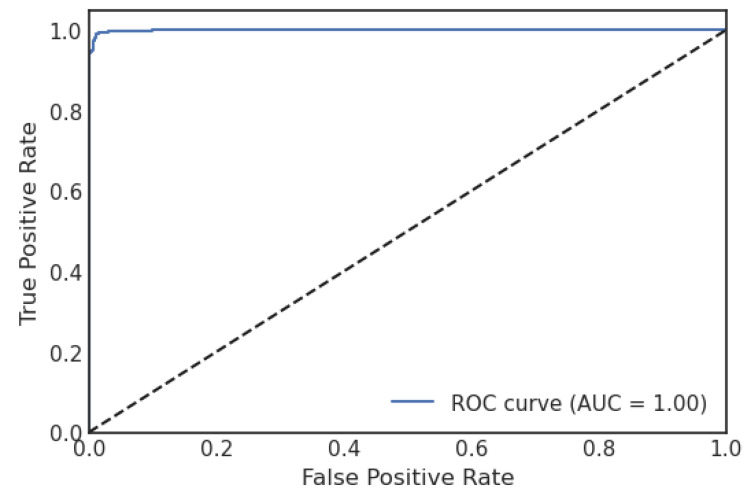
ROC curve of LDA.

Figure 6 shows the CM of the LDA algorithm of the proposed approach. It shows that if the TP and TN values are more significant than false positive FP and negative FN values, the interpretation of the proposed approach improves, and the model performs well on the used dataset. Figure 7 illustrates the proposed model’s ROC graph. The area of the ROC curve is 0.999%, which shows the model performed well.

**Figure 8:**
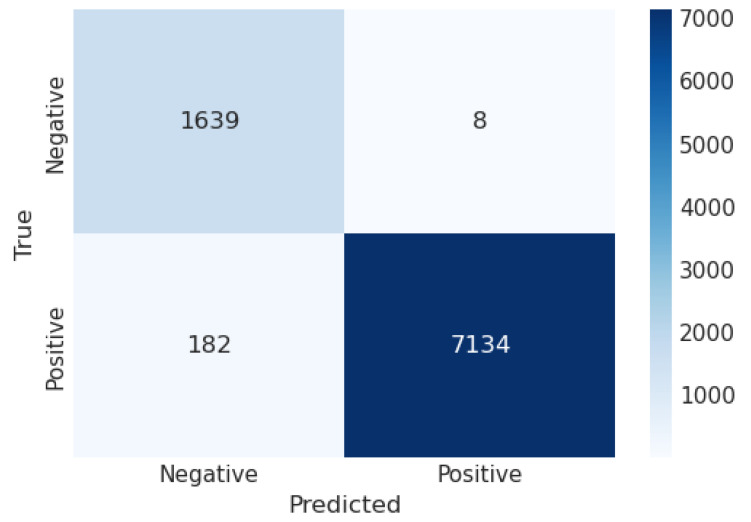
Confusion matrix of QDA.

**Figure 9:**
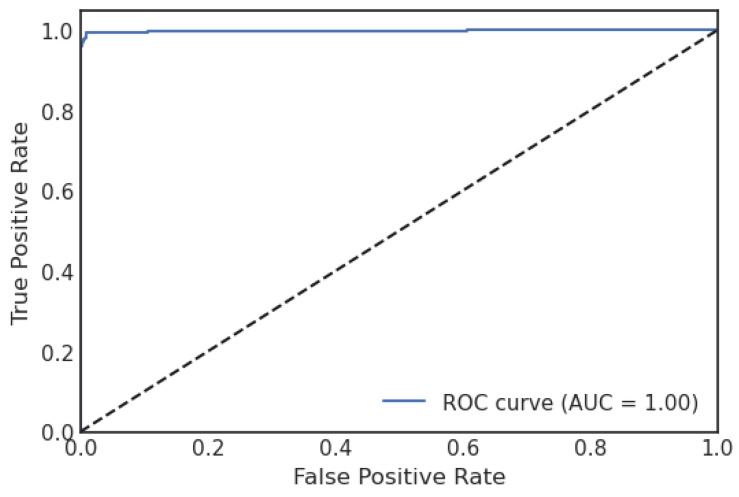
ROC curve of QDA.

Figure 8 shows the confusion matrix of the QDA algorithm of the proposed model. It shows that if the TP and TN values are more significant than the FP and FN values, the interpretation of the proposed approach improves, and the model performs well on the used dataset. Figure 9 illustrates the proposed model’s ROC graph. The area of the ROC curve is 0.997%, which shows the model performed well.

**Figure 10:**
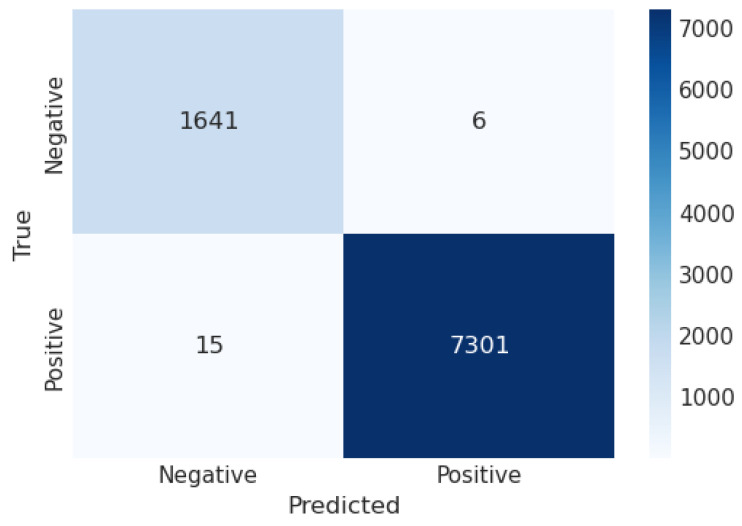
Confusion matrix of stacking.

**Figure 11:**
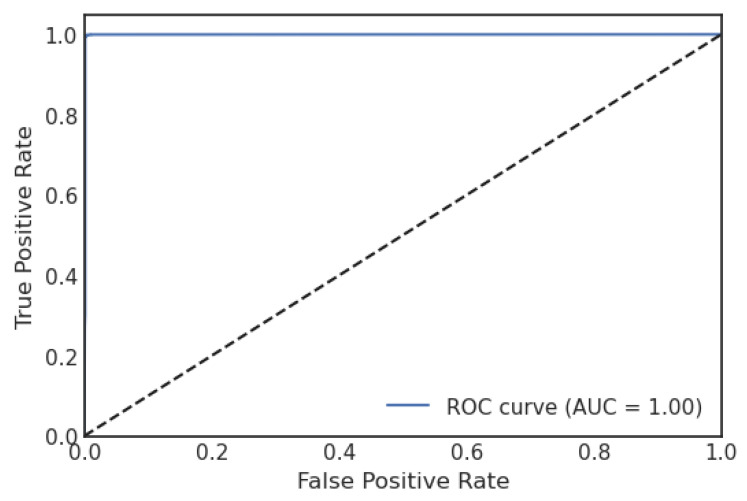
ROC curve of stacking.

Figure 10 shows the confusion matrix of the stacking algorithm of the proposed model. It shows that if the TP and TN values are more significant than the FP and FN values, the interpretation of the proposed model improves, and the model performs well on the used dataset. Figure 11 illustrates the proposed model’s ROC graph. The area of the ROC curve is 0.998%, which shows the model performed well.

Table 2 compares the proposed approach with existing approaches in stress detection using physiological signals. We compared the A% and F1% of different models on the WESAD dataset. As cited in the table, three existing approaches have been evaluated and compared with the proposed approach. Study [28] achieved an accuracy of 92.7% using an SNN model. In study [58], the authors attained an accuracy of 85.7% using a random forest (RF) model. Another study [38] achieved an accuracy of 87.7% using a CNN model, and in [59], the authors attained an accuracy of 96.26% using an ANN model. In comparison, the proposed approach achieved an impressive accuracy of 99.7% and an F1-score of 99.8% using a stacking model. These results further demonstrate the superiority of the approach in accurately detecting stress using the WESAD dataset.

The suggested method employs a stacking model with an F1-score of 0.998 and an accuracy of 0.997 to identify stress. The outcomes demonstrate that the suggested strategy performs better than the current approaches regarding accuracy and F1 score.

### 4.2. Discussion

To evaluate the performance of our stress detection model and mitigate the risk of overfitting, we adopt a cross-validation approach. Specifically, we employ k-fold cross-validation, dividing the dataset into k subsets or folds. The model is trained on k − 1 folds and validated on the remaining folds. This process is repeated k times, ensuring each fold serves as the training and validation set. The performance metrics, including accuracy, precision, recall, and F1-score, were calculated by averaging the results across all folds. By employing cross-validation, we can assess the model’s performance on multiple independent subsets of the data, reducing the likelihood of overfitting and providing a more robust estimation of its effectiveness.

To demonstrate the superiority of our method, we focused on the following aspects:

- Accuracy and performance: we compared our stress detection model’s accuracy, precision, recall, and F1-score with those reported in previous studies.
- Generalizability: We analyzed the generalizability of our model by considering the diversity and size of the dataset used compared to previous studies. A more extensive and diverse dataset can lead to improved generalization capabilities, enabling our model to perform well on unseen data.
- Efficiency: We assessed our method’s computational efficiency and resource requirements compared to traditional or previous AI-based approaches. This analysis demonstrated the practicality and scalability of our proposed method in real-world healthcare settings.

By conducting a thorough comparative analysis, we aim to highlight the strengths and advantages of our proposed method over traditional and previous studies. This will reinforce the significance and novelty of our approach to stress detection and contribute to the existing body of knowledge in the field.

## 5. Conclusions

In this research, we proposed chest-feature-based stress prediction on the WESAD dataset. The proposed model accurately determined stress using chest features from the provided data because of two reasons: we dropped fewer critical features and applied feature analysis, which included Z-score, feature selection, SMOTE, and normalization. The stacking model performs well with all machine learning classifiers, and the highest performance achieved regarding accuracy is 0.997%. The results for the chest set are better than for the wrist one. However, wrist sensors can be more easily integrated into real-life scenarios. We conclude that the proposed model can be applicable in everyday life and very useful in detecting stress states. Using this approach, we concluded that ML models could effectively define humans’ psychological and physiological states using data obtained from physio sensors. Our model correctly generated features from raw data, and after correctly selecting features, a suitable ML model can give a fairly good result. A disease is easier to treat the earlier it is identified. Medical professionals can detect stress more quickly and accurately with the help of the proposed method, which can spot these changes in people prematurely. However, it is essential to note that the proposed approach has only been assessed on the WESAD dataset and may not generalize to other datasets. Thus, the proposed approach will be evaluated on different datasets to determine the model’s generalizability, and a deep learning algorithm will be applied to the WEASD dataset.

## Figures and Tables

**Table 1:** Proposed model result: accuracy—A; precision—P; recall—R; F1-score—F1.

Model	A%	P%	R%	F1%
LR	0.978	0.998	0.975	0.986
LDA	0.955	0.999	0.945	0.971
QDA	0.978	0.998	0.975	0.986
Stacking Model	0.997	0.999	0.997	0.998

**Table 2:** Comparative analysis of the proposed approach with existing approaches.

Ref	Dataset	Model	Accuracy	F1-Score
[28]	WESAD	SNN	92.7	-
[58]	WESAD	RF	85.7	-
[38]	WESAD	CNN	87.7	-
[59]	WESAD	ANN	96.26	-
Proposed approach	WESAD	Stacking model	99.7	99.8

## Data Availability

Not applicable.
