# Correction: Almadhor et al. Efficient Feature-Selection-Based Stacking Model for Stress Detection Based on Chest Electrodermal Activity. *Sensors* 2023, *23*, 6664

**DOI:** 10.3390/s25247459

**Published:** 2025-12-08

**Authors:** Ahmad Almadhor, Gabriel Avelino Sampedro, Mideth Abisado, Sidra Abbas

**Affiliations:** 1 Department of Computer Engineering and Networks, College of Computer and Information Sciences, Jouf University, Sakaka 72388, Saudi Arabia; 2 Faculty of Information and Communication Studies, University of the Philippines Open University, Los Baños 4031, Philippines; 3 Center for Computational Imaging and Visual Innovations, De La Salle University, Manila 1004, Philippines; 4 College of Computing and Information Technologies, National University, Manila 1008, Philippines; 5 Department of Computer Science, COMSATS University, Islamabad 22060, Pakistan

**Reference Correction**

In the original publication [1], we incorrectly cited a retracted reference “Karthick, T.; Sangeetha, M.; Ramprasath, M.; Ananthajothi, K. Continuous Activity-Aware Stress Detection Using Sensors. *Wirel. Pers. Commun.* **2022**, *127*, 17.”. This reference has now been removed from the References. With this correction, the order of some references has been adjusted accordingly.

**Text Correction**

In Section 2.1, Paragraph 2, the following sentences were removed accordingly: “In [40], the authors present two experiments using a chest belt and a low-cost sensor for stress detection. They measured students’ mental stress one week before an exam and while using the internet. The heart rate and different aspects of the belt were similar to those examined during the device verification inspection, confirming the sensor reliability. Gold standard instruments were used for this comparison. With simple synchronization and data cleaning techniques, the authors chose extremely clustered, low-average information elements with the required chest data time for further analysis. The study’s primary goal was to examine tension throughout students’ college careers. Recruitment should take note of the results of pressure testing or stressing the student.”

The authors state that the scientific conclusions are unaffected. This correction was approved by the Academic Editor. The original publication has also been updated.
